# Mental health and psychosocial support among refugee children and adolescents with HIV in West Nile Uganda: An exploratory study

**DOI:** 10.1371/journal.pgph.0007000

**Published:** 2026-08-24

**Authors:** Molly Naisanga, Emmanuel Mpamizo, Noeline Nakasujja

**Affiliations:** 1 Department of Mental Health, Gulu University, Gulu, Uganda; 2 Department of Psychiatry, Makerere University, Kampala, Uganda; Durban University of Technology, SOUTH AFRICA

## Abstract

Refugee populations face disruptions in social services, particularly HIV- related care such as access to Highly Active Antiretroviral Therapy. Uganda hosts the highest number of refugees in Africa. These children and adolescents (CAs) are faced with several mental health and psychosocial challenges that are amplified by their humanitarian context. Multiple health and social welfare partners support Children and Adolescents with HIV (CA-HIV) in overcoming these challenges. However, no clear documentation exists on the mental health and psychosocial support (MHPSS) services available to aid the care-seeking process and design. We therefore set out to explore MHPSS services for CA-HIV in the refugee settlements of West Nile, Uganda. This study utilized an exploratory-descriptive qualitative approach. Participants included CA-HIV, their caretakers and people involved in offering MHPSS services to them. Altogether, we conducted 4 in-depth interviews and 9 focus group discussions. Study participants were recruited from refugee settlements in West Nile, Uganda and were invited to participate in focus group discussions, key informant interviews and in-depth interviews. The proceedings from these discussions and interviews were audio recorded, transcribed, and translated to English when necessary. Analysis by inductive thematic analysis was done using NVivo software. Three key themes emerged: Perceptions and markers of mental unwellness (including etiology, signs and symptoms), MHPSS and its related challenges among refugee CA-HIV (arising due to both the HIV serostatus and refugee status of the CA) and the role of the self, specialized and non-specialized service providers towards the MHPSS wellbeing of this population. MHPSS markers, challenges and interventions are multifaceted. The interventions involve specialized personnel, non-specialized personnel and the children themselves. All these ought to be carefully considered as we seek to address the contextual MHPSS challenges faced by refugee CA-HIV.

## Introduction

Refugee populations face disruptions in social services, particularly HIV-related care, such as access to Highly Active Antiretroviral Therapy (HAART) [1]. By the end of 2022, nearly 35.3 million people worldwide were refugees. Moreover, Uganda hosted most of these refugees in Africa, nearly 1.5 million of them, mainly in the West Nile region, of whom 58.9% were children [2].

Refugee Children and Adolescents (CAs) are faced with multiple challenges, particularly the subgroup who are HIV-positive, for whom the humanitarian context only aggravates their health challenges. The prevalence of HIV among refugee CAs in West Nile is unknown, though the national average of the prevalence of HIV among CAs is 5.1% [3]. HIV infection among CAs is associated with several problems including poor general health, stigma, discrimination, low self-esteem, delayed cognitive development and higher rates of depression [4,5], which are all worsened in a humanitarian context.

Refugees are heavily dependent on humanitarian organizations, and the pediatric HIV- positive population is no exception. The more specialized the service needed is, the more likely it can only be provided by these humanitarian organizations. There are about 44 partners, including 6 United Nations (UN) agencies, 27 international non-governmental organizations (NGOs) and 11 national NGOs as of June 2022 [6] operating within the West Nile region. These are in addition to the existing government structures, and several guidelines and policies to direct all of their interventions.

The government of Uganda released the fourth edition of the consolidated guidelines for the prevention and treatment of HIV and AIDs [7]. In these, a generic approach to Mental Health and Psychosocial Support (MHPSS) for HIV-positive people has been included, notably without specifics for contexts such as the humanitarian one. Therefore, further reference to the Inter-Agency Standing Committee (IASC) Guidelines on MHPSS in Emergency Settings is warranted [8].

The IASC MHPSS guidelines recommend a multisectoral approach highlighting the roles of the health and education sectors. Further still, these CAs will also rely on other institutions like religious groups and informal structures like family members and peers. However, the multiplicity of partners from various sectors has led to disjointed service delivery associated with poor service usage and adverse outcomes, specifically for CAs. Therefore, it is necessary to understand MHPSS clearly in the refugee context, including who does what, where, and when. We, therefore, set out to explore MHPSS for refugee Children and Adolescents with HIV (CA-HIV) in West Nile, Uganda.

## Methods

### Study design

An exploratory-descriptive qualitative approach was used. This approach was developed for health research about topics barely studied before [9], such as MHPSS services for refugee CA-HIV in Uganda.

### Study setting

This study was conducted in refugee settlements in West Nile, Uganda. The West Nile region is located in the North West of Uganda, bordering South Sudan and the Democratic Republic of Congo. The region comprises 12 districts. The refugee settlements were located in six of these districts. Some settlements were across district boundaries. Six refugee settlements (Bidi bidi zone 2 & 5, Rhino camp, Imvepi refugee settlement, Iriwa and Lobule refugee settlement) were chosen for participation using purposive sampling

### Study population

This study included refugee CA-HIV in West Nile, Uganda; their caretakers (these included the guardians primarily responsible for the CAs who were the parents or relatives to the child in other capacities) and people involved in offering MHPSS services to them (teachers, healthcare workers, community healthcare workers, police officers and refugee welfare council members).

### Eligibility criteria

#### Inclusion criteria.

Refugee CA-HIV, aged eight to 17 years and registered at one of the health centers in West Nile, Uganda; their caretakers, and people involved in offering MHPSS services to them.

### Exclusion criteria

- CA-HIV who have been receiving HIV care for less than a year and their caretakers.
- MHPSS service providers who have been involved in caring for CA-HIV for less than a year.

### Sampling size and procedure

Purposive sampling was used to choose participants, whereby participants were selected for their characteristics to maximize the variability of the sample and their potential to have information about MHPSS services in the respective settlements. Number of participants per settlement varied within a range of two to eleven.

For the CA-HIV, participants were purposively selected from the HIV clinic attendance records of the Health Center III or IV within these refugee settlements. These records were accessed in line with the Uganda guidelines for research in such vulnerable populations. As detailed under our ethics process, we obtained permission from the Gulu University Research Ethics Committee (GUREC), the Uganda National Council of Science and Technology (UNCST), Office of the Prime Minister (OPM), local government officials, health facility administrative officials who introduced us to and gave us permission to interact with the HIV clinic staff in charge of the records. Research staff only accessed these records in the presence of the clinic staff, and were not allowed to carry any details outside the designated office including hardcopies and photos.

If the Child or Adolescent (CA) deemed appropriate for study participation was a non- emancipated minor, their parent or guardian was informed about the study by their primary care provider and invited to consent for their participation. After which the CA was also invited to assent to participate. Furthermore, if the CA deemed appropriate for study participation was an emancipated minor, they were informed directly about the study by their primary health care provider and invited to consent to participate. We ultimately had 3 IDIs and 2 FGDs with the children

It is important to consider that FGDs raise ethical concerns related to privacy, confidentiality, and potential disclosure of HIV status in this population. We thus sought to engage children who were aware of their status. Further still, these children were quite knowledgeable of each other’s status as they had been attending care at the same clinic for at least a year as stipulated in our inclusion criteria. Also, in our setting several other partners operating in the settlements often brought the CAs together for various psychosocial activities and the CAs spoke of some of these activities during our data collection. In our assenting process we also emphasized confidentiality among the participants.

With these safeguards in place, we thought it pertinent to proceed with FGDs as they allowed us interact with several participants and gather a large quantity of data at once. They also gave us an opportunity to triangulate information we heard in the IDIs. The IDIs on the other hand allowed us determine personal experiences without the undue influence of group think which can be a challenge in FGDs of very similar populations.

The CA-HIV’s caretakers were identified as per their children’s records. Some of the caretakers also had their children participate in this study while the children of other caretakers were not interviewed for this study. This depended on the availability of other children whose caretakers had not been engaged, these would be preferred to maximize variability of views. Language was also a consideration for the FGDs and we aimed to bring together participants who spoke a common language. Further still, to maximize views, a caretaker could have only one child involved in the study, the most eloquent of the CA-HIV under their care. If a CA's caretaker was deemed appropriate for study participation, he/she was informed about the study by the CA's primary health care provider and invited to consent to participate. In total we had 1 in-depth interview and 2 FGDs with the caretakers.

Notably, the primary healthcare providers were critical to the selection of the CAs and their caretakers. The healthcare provider engaged for this exercise was based on guidance that had been provided by the administration within the facility. We aimed to work with the person who understood the participants best. The study and its goals were thoroughly explained to the healthcare provider to allow the purposive participant selection meet the goals of the study without undue discrimination and bias which would negatively impact study outcomes.

The MHPSS service providers who were health workers were selected from the health centers (that the children attended) following the guidance of the clinic's leadership. Other MHPSS providers were selected based on prior knowledge on how the MHPSS sector operates and information collected during interviews and FGDs with the CA-HIV and their caretakers. The CA-HIV and their caretakers helped identify the most notable MHPSS service providers who were informed and invited to participate in the study. In total, we had 5 FGDs with the MHPSS service providers.

Basing on our experience and reading, MHPSS within the humanitarian setting in West Nile is delivered using both the specialized (hospital based) and integrated approach (within other sectors). We thus sought to include the healthcare providers of the CAs, persons from the education sector in this case the schools, local leadership structures (Refugee welfare council members) who are often the gate keepers and liaison agents for many of the service providers and partners, the security personnel who often handle several psychosocial issues as part of their duties. Sampling was purposive and MHPSS personnel were recruited in liaison with our primary contact at the healthcare facility. Broad person specifications were discussed with the primary contact guiding selection. Invitation to participants was made by phone call and/or in person invitations.

Each FGD was comprised of at least 3 participants, following reviews [10,11] affirming that as few as three participants were acceptable for a successful FGD. Further still, the mix in languages was also constraining and allowed for only a limited number of people to be put together for an FGD. We had 33 participants in FGDs and 4 participants in IDIs. Details of the constituents of our 13 interviews are provided in Tables 1–3 below

**Table 1:** CA-HIV participants included in the study.

Refugee settlement	Health facility	Nature of interview	Participants	Gender and age of participants [Gender, Age]
Bidi bidi zone 5	Ariwa HCIII	IDI.01.CA-HIV	1	[M, 14]
Lobule refugee settlement	Lobule health center III	IDI.02.CA-HIV	1	[M, 16]
Imvepi	Yinga health center III	IDI.03.CA-HIV	1	[M, 16] – emancipated minor
Rhino camp	Omugo HCIII	FGD.01.CA-HIV	3	[F, 15] [M, 14] [F,17]
Iriwa	Dzaipi HCIII	FGD.04.CA-HIV	3	[F, 10], [F, 12], [M,12]

Note: M = Male, F = Female

**Table 2:** Caretaker participants included in the study.

Refugee settlement	Health facility	Nature of interview	Participants	Caregiving responsibility
Lobule	Lobule health center III	IDI.02.Caretaker	1	Mother
Imvepi	Yinga health center III	FGD.05.Caretaker	6	2 fathers, 2 mothers, 1 uncle, 1 cousin
Rhino camp	Omugo health center III	FGD.01.Caretaker	3	3 mothers

*Note: the caretakers present here consented to their child participating in the study where applicable. The caretakers were not physically present as part of the interview for the CAs, as this may have negatively interfered with the CAs’ participation.*

**Table 3:** MHPSS service provider participants included in the study.

Refugee settlement	Health facility	Nature of interview	Participants	Break down of participants
Bidi bidi zone 2	Yoyo HCIII	FGD.01.Service providers	4	Livelihood facilitator, HIV clinic head, teacher, HIV clinic counselor
Lobule	Lobule health center III	FGD.03.Service providers	3	Teacher, Police man, HIV clinic counselor
Imvepi	Yinga health center III	FGD.06.Service providers	4	Refugee welfare council (RWC) chairperson, HIV clinic counselor, teacher, facility psychiatric clinic officer
Rhino camp	Omugo HCIII	FGD.02.Service providers	4	Community health worker, teacher, facility psychiatric clinic officer, RWC committee member
Iriwa	Dzaipi HCIII	FGD.07.Service providers	3	Case management assistant, Health center incharge, HIV clinic counselor

### Data collection procedure

Data was collected using IDIs and FGDs. Participants were invited to a private and secure place within the health centers for the interviews. Each interview lasted about 1–2 hours and a semi-structured interview guide was used. We had separate interview guides for the CA-HIV, the caretakers, and the MHPSS service providers. For each of the 3 populations we had separate guides for the FGDs and IDIs, with same concepts though variations in wordings based on what may be appropriate in a group and what may not be appropriate.

These guides were developed based on our experience and literature in the field of MHPSS within refugee settlements. The FGDs aided the rapid discovery of collective perceptions of MHPSS services for CA-HIV in West Nile, Uganda while the IDIs allowed us explore other issues that could not be explored in a group setting. IDIs also allowed us to interact with individuals who could not be interviewed in a group due to the variances in language among the refugees. All interviews and FGDs were conducted by the principal investigator (PI), with the help of a translator, when using a local language was necessary. Interview and FGD proceedings were audio recorded and field notes taken in a notebook. Data was collected from 16^th^/October/2024–7^th^/November/2024.

Interviews were conducted till it was determined that saturation had been attained. That is to say, no substantially new information and insights relevant to the study were emerging from the participants.

### Data management

Each IDI and FGD was assigned a unique identification number given to the audio file and related transcripts. All data related to the research, including audio recordings, transcripts, and informed consent forms were stored in a secure and safe place, only available to the immediate study team. Enrolment forms recorded participants’ names, socio-demographic details and corresponding identification numbers were kept separately from other data files. All recorded and written records only displayed participants’/group identification numbers, not individual names.

### Data analysis

Data was analyzed using inductive thematic analysis; the final transcripts were transcribed, translated, cleaned, and read several times to gain familiarity with the data. Text blocks in the transcripts with information relevant to MHPSS services for CA-HIV in West Nile, Uganda, were highlighted. Initial codes were generated based on these highlighted blocks. Several codes were merged with others to form the final set. Related codes were grouped into themes.

Then the themes were reviewed, refined, and defined. Quotes were also extracted to support the interpretations of these themes. Nvivo version 15.5 software was used in this analysis process.

#### Quality assurance and control.

The study tools (information and consent forms) were translated into Juba Arabic, Bari, and Dinka languages to accommodate participants who preferred communicating in those languages. The audio recordings were also compared with their transcripts to verify transcription accuracy. To ensure trustworthiness, we applied Lincoln and Guba’s trustworthiness criteria [12] as detailed below.

Credibility (confidence in the truth of the findings) was enhanced through prolonged engagement in the field spanning over three weeks, triangulation of data sources by including three distinct participant groups (CA-HIV, caretakers, and MHPSS service providers) using both FGDs and IDIs. Transferability (applicability to other contexts) was addressed through thick description of the study setting and participant characteristics. We provide detailed contextual information about the West Nile region, the humanitarian service landscape, and the dual identity (refugee + HIV-positive) of the study population, enabling readers to assess the relevance of our findings to similar settings.

Dependability (consistency of the findings) was ensured through a transparent and auditable research process. We maintained a comprehensive audit trail that included raw data (audio recordings and transcripts), field notes, coding frameworks, and records of all methodological decisions (e.g., sampling approach, language translations and analytic steps). This trail allows an external reviewer to follow the research process from data collection to theme development.

Confirmability (neutrality and grounding of findings in the data) was enhanced through ongoing reflexive engagement. As the principal investigator is a Ugandan citizen (not a refugee) and a psychiatrist, we consciously acknowledge that clinical training and positionality as a Ugandan national (interacting with refugees) might influence the research process. This might have predisposed the team towards medicalized interpretations of distress whilst misconstruing participants’ experiences that might have been unfamiliar to her. To mitigate this, we deliberately grounded all thematic interpretations in participants’ own language and direct quotations as depicted in the results section. Further still, the principal investigator also engaged in conscious bracketing (actively setting aside her assumptions) during interviews and analysis.

### Ethical considerations

Acknowledging that talking about HIV/AIDS may be traumatic to some of the participants, the PI, being a psychiatrist provided brief counseling to those who were mildly distressed through the course of the interviews. After that, if necessary, the affected participants were referred to the nearest government-owned health facility with mental health professionals, where they could receive more care free of charge.

Ethical approval was sought from the Gulu University Research Ethics Committee (GUREC-2023–776) and the Uganda National Council of Science and Technology (UNCST), research registration number- HS4486ES. Permission was also sought from the Prime Minister’s office and each of the proposed research sites before the commencement of the study. Written informed assent for participants younger than 18 and consent for adults was obtained from the participants before participation.

Participants were able to opt out of the study freely and their decision to proceed or not to proceed with the study did not influence the kind of treatment they received thereafter. Each participant was given a token of appreciation for their time of 20,000 UGX (≈ 6 USD). This was given out at the end of the interview. Transport reimbursement of 20,000 UGX (≈ 6 USD) was also provided for each participant. Thus as a standard, each participant received 40,000 UGX (≈ 12 USD) in total.

## Results

A total of 9 FGDs and 4 IDIs were conducted, with a total of 37 participants. Some representative quotes from these interviews have been presented in this results section. Each quote is labeled with the nature of interview (FGD/IDI), the identification number, and the nature of interviewee (CA-HIV/Caretaker/Service provider). Three key themes emerged from this study: perceptions and markers of mental unwellness, MHPSS and its related challenges among refugee CA-HIV and the role of self, the specialized and non-specialized service providers towards the MHPSS of CA-HIV. A summary of these findings is provided in Table 4 below.

**Table 4:** A summary of themes and subcategories from this study.

Major theme	Subtheme	Subcategories
Theme 1: Perceptions and markers of mental unwellness		Definition of mental health and causes of mental unwellness
		Signs and symptoms of mental unwellness
		Suicide
Theme 2: MHPSS and its related challenges among refugee CA-HIV	Challenges faced by refugees	Inadequate food
		Other livelihood issues
		Refugee status and settlement issues
	Challenges faced by children due to the HIV diagnosis	Challenges of disclosure and accepting diagnosis
		Issues around access and use of medicine
		Issues around adherence
		Experience with stigma and/or discrimination
Theme 3: Role of the self, specialized and non-specialized service providers towards the MHPSS of CA-HIV		Healthcare facilities and their attendant processes
		Involved collaborators and service provision partners
		Local leadership structures and schools
		Family
		The Self

### Theme 1: Perceptions and markers of mental unwellness

#### Definition of mental health and causes of mental unwellness.

The participants argued that it is important to understand mental health, even before any discussion on the topic can commence.

*…most of these dialogues, me what am always aiming at because I want people to understand mental health, because the first solution is for someone to know that mental health exists. So, most of my dialogues, actually most of my topics have been about talking to people, telling them what mental health is […] so that people first understand what we are dealing with. But maybe as time goes on, they will be able to handle the other topics. (FGD.02.Service providers)*

In this section participants were engaged on broader aspects of mental health beyond just the population of study though aspects pertaining to the population of study were emphasized as much as possible. The definitions of mental health were many, reflecting the broad variations in participant categories. Definitions mainly focused on the involvement of the mind, while others stretched to involve economic, spiritual and physical aspects. Definitions were also presented as the presence of something like ability to cope, working brain or the absence of some things like stress or problems.

*A state of being physically, mentally and socially well-being of an individual in terms of his mind (FGD.06. Service providers)*

*[…] it is the reasoning capacity of a human being. How we reason, whether a person is reasoning good things or bad things [….] (FGD.05.Caretakers)*

*Interviewer: So when someone has a problem in their head, how are they?*

*Respondent: Sometimes they cannot feel okay. They can feel just like they are having stress. (IDI.03.CA-HIV)*

The state of mental unwellness, as explained by the participants could be caused by congenital issues, infections, drug abuse, too much stress and contextual factors (like disasters in the country and domestic violence at home). Certain factors like curses, generation to generation issues were also mentioned to have a role in the etiology of mental unwellness.

*They are running mad like that due to HIV. Because once when you were tested HIV positive and the counselor has not done enough, at times the counselor could do enough counseling but still that thing [testing HIV positive] can torture you. It can stress you and at the end you run ‘mad’. (FGD.04.CA-HIV)*

#### Signs and symptoms of mental unwellness.

Once mentally unwell, the symptoms varied to include abnormal reasoning (low understanding in class, answering in a contrary manner, declining performance, forgetfulness), abnormal behavior (talking alone, uncoordinated talks, poor hygiene, walking naked, among others). Changes in functioning manifested as refusal to attend class, missing hospital appointments for a previously regular client may also signal mental unwellness. Mood changes (emotional outburst and mood swings) and other mood related aspects that impact socio-occupational functioning like abusing others, aggressiveness and isolation may also occur.

*For example, in our setting in the school there if the child has mental illness, you find that if you are teaching, the child takes long to understand (FGD.02.Service providers)*

*Interviewer: [….] when you hear someone is mentally unwell […], how would those people behave?*

*Respondent: They be dirty, others move naked, others talk alone (FGD.04.CA-HIV)*

*Interviewer: [….] how do you identify children who are having mental health challenges?*

*Respondent: [….] when you see a child is sitting down, shy, isolating himself from the rest of the colleagues, definitely this child is having problem, is having challenges. (IDI.02.Caretaker)*

#### Suicide.

One aspect of mental unwellness worth special mention that emerged during the course of this study was suicide. The participants stated that aspects of mental health can include suicide or killing. Participants shared testimonies of various people who had died by suicide including, adults and children, refugees and nationals, those who had HIV and those who did not. The service providers stated that suicide could also present in the form of non-adherence to medication which was not present before, as the intention of these non-adherent patients sometimes was death and this could only be found on further probing. Many participants, including the children presented the management option of counseling, from them to someone in their midst who may have wanted to die by suicide

*INTERVIEWER: [...] So, if someone thinks about killing themselves what should they do?*

*RESPONDENT: So, what I will do is I will either go and remove him out from the rope or I have to encourage him or give him counseling not killing. (FGD.01.CA-HIV)*

Other presented solutions included involving: the church, police, local government structures like the Community Development Officer and relocating the person. In as much as one mental health specialist argued that all patients who have attempted suicide should be brought to the facility, the role of the health facility as shared by the caretakers included giving tablets or solely treating wounds that may accrue from the method used to attempt suicide.

*INTERVIEWER: What about the hospital? Does the hospital have a role to play in this kind of situation?*

*RESPONDENT 1: A person who wanted to commit suicide, unless if there is evidence or a proof that this person or a remainder of the rope that has left in the neck, something that shows that this person has really tried or attempted to commit suicide that is when you refer the person to the health facility. You bring the person to the facility and then maybe the health worker has to do her part […]*

*INTERVIEWER: So, what about (Respondent 3), what's the role of the facility? You said, if there's evidence, like the rope you now bring to the hospital. So, what are your expectations of the hospital at that point?*

*RESPONDENT 3: The role of the health facility is only to give some first aid in case there's a wound. (FGD.01.Caretaker)*

### Theme 2: MHPSS and its related challenges among refugee CA-HIV

At the onset of this section, it is important to note that the MHPSS challenges mentioned in this section may not necessarily be exclusive to the population of study, but were presented as lived experiences by refugee children with HIV, lived experiences or observations of the caretakers of these children and as observed by the service providers of these children. The population of study possesses a dual identity: children who have HIV and are refugees: and this section is approached under those subthemes.

The caretakers were fully cognizant of their personal HIV statuses and that of their children, they often times also looked after many other children who were not HIV positive. Further still, they also had other aspects about their identity that defined them, for example being refugees and being secondary caretakers and not the biological parents of their children in some instances. Though a unique population, visibility was not very welcome.

Some special populations mentioned, had received special recognition and specialized mental health packages like the deaf and blind people taught by Save the Children and Health and Inclusion (HI) (FGD.01.CA-HIV) and one might think the refugee children may as well benefit from similar specialized services but this was not the case. The service providers’ perspective depended on the setting. If they knew the HIV status of the children (like the HCWs) then that variable was factored in as they spoke about them. If they did not know the status, then they considered the population of study just like any other refugee children.

### Challenges faced by the refugees

#### Inadequate food.

Through the course of this study, it was glaringly clear that food was one of the most if not the most pressing challenge for this population. The food ration (food material or in form of cash) that the refugees receive had been reduced significantly over the course of time. In some homes, where these children resided, even when this little food was present, the children were denied food by the caretakers. One of the reasons given was that they were not biological children of their present caretakers. Others who were willing to dig their own food had failed due to lack of land or if the land was present, it had reportedly been found to be barren and rocky. For those who overcame all these challenges and still planted, the unpredictable weather had gone on to scorch their plants. To solve this problem, some homesteads engaged in casual labor (like digging for other people) to try and source some food.

When the service providers within the health sector dealt with our study population, they had often tried to solve this food problem by referring the clients to partners who were equipped (at least on paper) with the resources to manage this problem. Unfortunately, this approach had reportedly not been very fruitful. One partner represented in this study reported that they gave knowledge on growing food, they were not offering seeds, another partner was offering that. For our specific population of study, the matter was worsened by the alleged fact that the HAART drugs not only had to be taken alongside food but also significantly increased the food consumption rate of the children.

*Refugees normally, you know, the side effects of these drugs, some of them they need a lot of eating. Sometimes now we have a saying that we think these drugs they [the children] are taking also goes inside and eat the food that you have eaten. Why are they feeling always hungry like that? [...] When they don't take the drug, they don't feel hungry. But when they take the drug, their hunger level increases. That means the drug might also be eating the food in their stomach. So, we realized a child of that kind may be having attention or concentration issues in class. If the teaching staffs are not aware of it, might end up going to beat this kind of child. (FGD.06.Service providers)*

*In addition to these major health HIV related conditions, they [the children] have issues. You know, taking drugs daily on top of the education is part of the problem they are facing. All these drugs need some kind of food, in most cases, food is not constant and you end up taking it in an empty stomach, it is a big challenge. (FGD.07.Service providers)*

As a matter of fact, when asked about how they could be supported to improve their mental health and psychosocial wellbeing, the children were quick to note that providing food such that they do not have to think about what to eat would be very helpful (FGD.01.CA-HIV)

#### Other livelihood issues.

Besides the challenge of food, the children faced other challenges. These included the outright lack of some items like shoes, mattresses and clothes. There had also been the challenge around accessing any help that one may benefit from by virtue of their HIV sero status. The need to maintain the secret of one’s HIV status led to the challenge that certain opportunities for help were missed.

*So, even though you are hungry, there is nobody, you cannot say I am hungry because I was sick, ABCD, help me with some posho so that I will eat, I will have the energy. So, really, in the villages, in our communities here in [this refugee settlement], to get this support, it is a problem because we are just hiding the other statement. You cannot make it openly to be supported. Unless if there is some organization like health centres, those who know us just secretly, [...]. For instance, for us, who has disease sickness, there is no other support, because we are hiding things, we cannot make it open. (FGD.05.Caretakers)*

Also considering that any help or support would usually come through the local leadership structures, some Refugee Welfare Council (RWC) members had been noted to avail this support to their own acquaintances, leaving out the persons with HIV for whom this support was meant. For the few aid items that reach the recipients, it had been noted that many beneficiaries just went on to sell these such that they could use the money for other issues that were more important to them at the time. The other challenge that had presented itself with aid giving was that some parents had fallen into a complacency state expecting various organizations to take on the entirety of their obligations (like providing books) as parents for these children. Finally, some modalities of aid remained a mystery for the beneficiaries. Lack of understanding about who is receiving what and why, had produced a sense of discrimination within the communities.

*These ones they are [particular organisation] supported. You find that from my country South Sudan when it comes support is given general not segregation. (FGD.01.Caretakers)*

*[...] the facility can provide rice, beans and food that can be given to us so that the children have a healthy growth. Because some of the support is given to other specific people and why is it that we are not supported, especially these ones who are HIV positive […] my son is here, at the facility. But now, as we are getting the service here, some of the clients who are also HIV positive, they are supported and we are not supported. So, I don't know exactly what is the problem. (IDI.02.Caretaker)*

#### Refugee status and settlement issues.

The movement from one’s land of origin to this land was noted to sometimes be accompanied with bad memories (of war and guns) as was back home and significant economic losses (among other losses) associated with the transition, both of which could be significant stressors. The present areas of stay (the settlements) had been noted to be muddled with significant inter-tribal clashes that had necessitated community wide psycho-social interventions.

*Also, the reason why they brought the issues of talks is because there are a lot of tribes in the camps. So, like in Iriwa, we're mixed up, we have [tribe 1] from South Sudan, we have the [tribe 2]. So those people [tribe 1 and 2], they look for problems, that's why they brought those things of talks, among the refugee settlements. (FGD.04.CA-HIV)*

Further still, the settlements had been found unsafe at times, with the need to institute curfews for safety. Reportedly some people had lost their lives in the hands of certain youths who were *‘behaving like rebels’* engaging in undesirable behavior like raping, smoking and even killing people at night.

### Challenges faced by children due to the HIV diagnosis

#### Challenges of disclosure and accepting diagnosis.

To start with, the time preceding disclosure, as reported mainly by the caretakers was one filled with a lot of questions on the part of the children mainly on why they are taking the drug. When disclosure was achieved (something rather difficult though the healthcare workers greatly encourage the caretakers to do), immediate reactions on the part of the children were marked by denial of status, crying, short lived distress or outright refusal to take the medicine. This revelation was described by one of the children as a loss of *‘freedom’* from what they had before this information was revealed to them. This stress about the diagnosis was also accentuated by the wider community perceptions of the illness. Some caretakers were noted to refuse to take the children to school on learning about the children’s status. In the eyes of some community members, the disease was viewed as an immediate cause of death, an assertion they were not shy to let the children know about.

[…] *so, when my grandmother didn’t told me, I stays free, I have no problem. But now, after realizing that I have that disease, now that is when I put serious it in taking the drugs. I am taking the drugs, though I have stress […] I think I will die [...] because people talk that if you have this sickness you don’t last for long definitely you will die. (IDI.01.CA-HIV)*

Beyond the children, many others in the circles of care for these children felt that making them aware of their status would help them to take better care of the children.

*Sometimes when a child [who has HIV] may feel sick, you know very well this is the sickness of this child. It would be easy for you to identify the child to the health center. (FGD.01.Service provider)*

#### Issues around access and use of medicine.

As expected, the HIV positive children are meant to be on lifelong treatment. As reported, not all facilities (sometimes those closest to the refugees) had this medicine. The children (with or without their caretakers) have had to move long distances to access this medicine. One of the caretakers reported having to carry their 10-year-old, sometimes, for some bits of the long journey. Despite the long distances and the exorbitant transport costs they may attract, some clients were insistent on the far placed health facilities citing personal comfort at these particular facilities. At the facility, it had been observed that some age groups of the children (reportedly 10–18) were often absent for their reviews, being represented by their caretakers.

The children who got the drugs often found difficulty in adhering to their medicine due to the lack of food (as earlier discussed). Other areas of care like follow up reviews were significantly hampered by bad weather (rain or too much sunshine) and a patchy telephone network where the clients resided making it difficult for the healthcare workers to reach them.

*[…] that facility [the one closest to us] is not well constructed [...] So these drugs are not in that facility. So that’s why I decided to come here. […] Sometimes we come using motorcycle, sometimes we have to move from [name of place] and when the child gets tired I have to carry him. (IDI.02.caretaker)*

*For us at our level here, the major challenge we are just facing at the facility level as service providers, is only majorly the challenge of the hostile environment. Sometimes hot, sometimes rain (FGD.06.Service providers)*

#### Issues around adherence.

It was reported that the children, unlike the adults needed follow up as they sometimes *‘dodged’* their doses. The health facility workers thus, had found it useful to often follow up on these children, sometimes even necessitating home follow up visits. Incentives like giving plumpy nut (Ready-to-Use Therapeutic Food, RUTF) alongside the HAART medicine had been found to aid compliance only that the presence of these was erratic. At one of the facilities, they reported to have just received plumpy nut which was soon to expire. Indeed, in the past it was noted that when the RUTF project which had been ongoing was stopped, some children refused to take their medicine and turned unsuppressed. The children also sometimes found themselves with unsupportive caretakers who worsened their adherence despite strong adherence counseling often availed to the children.

*[…] we have like he said that girl, she is really facing a lot. The aunt is torturing her […] Even sometimes she doesn’t take drugs [HAART]. Last month it took her two weeks without taking. The aunt refused her to take her drugs. Now I decided to give her medicine to this expert client of ours. Every morning, he goes there to give her the drugs, it is a bit hard (FGD.01.Service providers)*

### Experience with stigma and/or discrimination

Some of the children had been advised not to share with other people information that they have HIV. The experience had been that people laughed at you when they found out that you had HIV or used derogatory terms like referring to the children infected with HIV as ‘*moving dead bodies’*. Further still, it was reported, that once people found out that a child had HIV, then they did not want to associate with that child. Parents would even go the extra mile to ensure that their HIV negative children never played with the child who had been reported to be HIV positive

*Another issue is, like for me I have girlfriends, this one, she has a fellow girl, so now girlfriends, whereby in our free time we stay together, we play together. So, whenever I am free, I feel like to go and play with my fellows. So, I could go, after reaching there, the parents of my fellows could say, why are you coming here? You want to infect my children, you want to infect my daughter, you want to infect my children, no? Go away, go back, so they could chase me away. (FGD.04.CA-HIV)*

The children thus cannot interact freely in the community, they are afraid of moving in the community, cannot engage in activities they love like football, and others had gone on to refuse to go to school. One child’s plea was that they should provide a specific place for only people with HIV, and also a school for only children with HIV. On a positive note though, the children reported that the segregation they experienced in Uganda was less than the one experienced in their countries of origin and they attributed this to the sensitization of the communities on HIV which is stronger here. Also many of the service providers and family members were noted to be a constant source of comfort and encouragement in the glaring face of this challenge.

### Theme 3: Role of the self, specialized and non-specialized service providers towards the MHPSS of CA-HIV

#### Healthcare facilities and their attendant processes.

At the top of the MHPSS service provision pyramid is the healthcare facilities and the processes involved as the healthcare workers (HCWs) attend to this population (CA-HIV). The role of these was greatly acknowledged by the children who reported a comforting environment as had been provided by the HCWs.

*We are feeling that since we started taking medication from this facility, we are always feeling at home whenever we come for refill [...]We are always feeling okay because the health workers at this facility, especially at the HAART clinic, they know how to talk to people. They know how to talk to us. And they always advise accordingly most especially about how to take medication (FGD.02.CA-HIV)*

Besides HAART refills, the HAART clinics were also engaged in Sexual and Gender Based Violence (SGBV) services and channeling of food support to the participants. Notably, some facilities had specialized mental health personnel while others did not have, in all cases screening and referrals were highly encouraged. Some facilities were able to admit the mentally ill, only referring those who were severely unwell. There were also specialized partners who received clients for particular services (like partners serving the physically disabled, partners offering specialized psychotherapy services), though referrals were as is typical of the Uganda healthcare system with each health facility referring to another above it as specified in the health facilities’ ladder.

In as much as referrals were greatly emphasized for non-mental health professionals (teachers, livelihood supporters, RWCs), the need to empower them with the skills to identify children who were mentally ill could not be overstated. Further still, they (the non-specialized MHPSS service providers) emphasized that even when referrals were made if the referred were not initially counseled, chances that they would not follow through with the referrals made were rather high. Hence the need for basic counseling skills on the part of these non MHPSS specialized work force. Interestingly, concerning the referral chain, the patients and their caretakers hardly knew about it and those who knew about it hardly understood or trusted it.

*They told us, when you have found the child like that, you are to report [to the health center]. And when we send those children, others absolutely will not go... You cannot even know whether the child was assisted or was not helped. (FGD.01.Service providers).*

All factors notwithstanding, linkage and referral forms for psycho-social and other needs provided by the government as part of the health records system were readily available and commonly used in many facilities. On the other hand, other tools particularly those proposed by the Ministry of Health guidelines on HIV management to be used for screening for mental illness in HAART clinics (like the PHQ9, SRQ20, SADPERSONs) had largely remained theoretical. Some healthcare workers had heard about them, while to some they were completely a new phenomenon introduced by the interviewer. On a high note though, one facility had taken the initiative to print some of the forms and pin them on the wall such that they could easily be accessed.

*…we have printed out the one for screening GBV, we have also printed out, only that one for screening for depression, that and so on, we are not printing them out. But we have printed those pinned on the wall […] So, you can always just even glance at it the client may not know that you are looking at that thing […] (FGD.06.Service providers)*

Besides the tools screening for mental illnesses within the HIV population, other steps towards integration of mental health care in HIV care reported by the participants included the routine direct enquiry on MHPSS wellbeing of their clients; integrating mental health into other services like relief item distribution and sexual and reproductive health services; trainings on mental health- though these were often integrated trainings that combined several other topics for example topics on other non-communicable diseases. Integration also meant that if a child had mental illness and HIV then they should be seen in the same clinic on the same day. As a matter of fact, in one facility it was reported that the HIV clinic and mental health clinic shared a room and this really facilitated this process. Adaptation to this new model (of integrated HIV and mental health care) was still a process for the clients as the comprehensive services now meant that the reviews took relatively longer than they used to.

Still linked to the health facilities, were the young people and adolescent peer supporters (YAPs) who did a tremendous job of talking to and following up on these children and adolescents with HIV. Another category of service providers were the partners who were notable for doing out reaches and dialogues on mental health in the communities. These dialogues were often organized depending on identified needs, with mobilization of the community done in liaison with the Refugee Welfare Councils (RWCs). The dialogues were often conducted in the community halls or within suitable communal meeting places within the communities. These outreach efforts were riding on existing sensitization efforts like those organized by the Office of the Prime Minister which usually gave talks on different topics- MHPSS and non MHPSS topics. Notably, the topic of MHPSS among refugee children with HIV or just MHPSS among children had never been explicitly addressed among all the participants we engaged.

#### Involved collaborators and service provision partners.

Working with, within or even outside the health facilities were a myriad of partners whose combinations varied from location to location. The partners included United Nations (UN) Agencies and several Non-Government Organizations (NGOs). The MHPSS roles of the NGOs varied to include those that had been involved in training on various MHPSS topics, to those that had supported aspects that are relevant to MHPSS like food, livelihood, returning pregnant girls to school, conducting community dialogues and outreaches, among others. Majority of the partners that were discussed during the course of our interaction with the participants were involved in multiple sectors, with health being one of their many other activities.

Notably, the partners though numerous had not been able to solve or only partially solved the many pressing issues the participants had. As a matter of fact, support was declining by the day particularly in the area of food. The partners seemed to run projects that were time bound, often times leaving the beneficiaries without good replacements once projects were terminated.

*There was a time, I think it was in 2014 or 2015. This organization called [name of organisation]. These people were supporting these children. Every after one quarter, they are calling for a meeting. You talk with them, you share the experience they are facing. And you also talk to them, their understanding. But when they left, there is no organisation which can pick up like [name of organisation] to support them. (FGD.07.Service provider)*

#### Local leadership structures and schools.

These were credited for the supportive role they had played in the MHPSS wellbeing of many refugee children with HIV. Some of the people in these structures were among the few people outside the healthcare workers and the children’s families who had been made aware of these children’s HIV serostatus. This is information the local leadership had got to know through procedures of support distribution like giving out food. In as much as some children were uncomfortable with this fact, MHPSS support in form of counsel and comfort in times of need from these local leaders was reported by the children.

*So, with the LC, one day children were playing and I was down. I sat down and then when the LC saw me that I sat down he called me. So, I went and the LC advised me that I should really not think too much. I should join the other children playing. I should not think a lot (IDI.02.CA-HIV)*

Indeed, other specialized personnel reported empowering the RWCs as they were deemed important for the MHPSS wellbeing of these refugee children with HIV. Another group of local leaders relevant to this topic were the cluster/block leaders, these were credited for gathering the children and talking to them about various topics including management of stress. The cluster leaders, reported the children, could also be approached by anyone who felt the need to talk.

Schools were pivotal to the wellbeing of these children, and service providers had put in effort to encourage the children to go to school as they sometimes wanted to drop out due to their HIV serostatus. The children reported support from head teachers and other teachers. This support was remarkable for positive words of counsel encouraging the children to focus on school and to be helpful to their parents. To aid this process, one of the schools represented had improvised with a counter book that the headmaster used to record all pupils with all their important details including MHPSS issues. Other players were also directly involved with schools pertaining to the provision of MHPSS: the health facilities often organized school talks for the children, other IPs like Transcultural Psychosocial Organization (TPO) also directly worked in the schools offering MHPSS services to the children who needed them as identified by the school authorities.

#### Family.

The children reported tremendous support from their families. This was often provided by various family members including grandmothers, parents, aunties and siblings. This support presented in a variety of forms like accompaniment to pick the drugs, counsel on first discovery of diagnosis and support in the face of stigma and discrimination from other people. The caretakers reported receiving advice on how they can support the refugee children with HIV under their care. Further on this support, the families of the infected children had benefited from home visits by personnel from the health facilities. These home visits were sometimes a result of probable issues at home reported by the children, missed appointments for drug refills, or outright initiatives from the facility personnel to conduct impromptu home visits to check on the children and allow the HCWs fully appreciate the child’s situation.

*One way of identifying these children is through screening. We do psycho-social screening during the time when they come for their refills. Some of them we also end up identifying them when we do home visits. There are some unplanned home visits, where you just stumble home. Sometimes, you find that you just find different issues. You know, some of the parents are the ones also inflicting these issues on these children. So, when we go there, unplanned, we may find different issues. (FGD.06.Service providers)*

Some partners have supported the home visits by providing cash tokens, to allow the facilities conduct this important exercise.

#### The self.

Finally, the children also employed multiple coping mechanisms to help them deal with the existing MHPSS challenges. Some of these were reiterated by the service providers indicating the power of passing on knowledge and empowering the children. These varied from relaxation exercises, reading books, watching movies, playing football, joining good groups, moving with friends and moving to church. Notably, some of the suggestions put forward are negated by stigma, that is to say, you cannot join a group if no one wants to play with you in the first place. In action points, the children recommended that there is need to provide games and items of play like balls for instances when they gather, and the need to address stressors like lack of food. It was also suggested that children need to be trained on how to control their emotions.

*They said about, if you are just feeling you are stressed, there are other things you can do when you are stressed. Also they talked about breathing, where you sit on the chair, on the chair while you breathe in and out, to make you relax from that stress (IDI.03.CA-HIV)*

*Interviewer: […] what are some of the things that children who are refugees, they have HIV, what are some of the things they do to help them deal with stress, with too many thoughts and worries when they are not feeling well?*

*Respondent: I prefer playing football but sometimes they refuse me, I can’t even explain the reason.*

*Interviewer: They refuse other children?*

*Respondent: Yes, to play with me (FGD.04.CA-HIV)*

## Discussion

Wide variation in perceptions and markers of mental health ranging from disease and medical model to social and spiritual beliefs is a finding resonant with findings from other scholars [13]. Pertaining etiology, biological, psychological and social issues were ably alluded to in this study as is the widely held explanatory model for mental illnesses [14,15]. In as much as identifying mental health problems in people under the age of 18 years can be challenging since presentation of mental illness in this population maybe subtle [16], several signs and symptoms of mental illness in the refugee children with HIV and other populations were elaborated in this study to include challenges with school, behavior and mood disturbances. These disturbances in functioning and social interactions reflect a concept underlying most if not all mental illnesses: socio-occupational derangements [17,18].

Considering the dual identity of the population under study, the MHPSS challenges were many. The challenges related to the HIV status found in this study have indeed been studied broadly. Disclosure to a child about their HIV status has been observed and reported by others in the literature as a significant stressor [19–21] and this study was no exception. HIV positive status is further complicated by stigma, HIV remains a highly stigmatized disease in sub-Saharan Africa [22,23]. Indeed, in as much as we are grateful for the life-saving treatment in form of HAART, both access to the medicine and adherence to the treatment remains a challenge [24].

The positive HIV status of the children is further complicated by their refugee status. As reported by our participants and supported by other scholars, besides the difficult socio-economic circumstances due to the widespread cuts in food rations and other forms of support towards livelihood [25], the refugee settlements are also rather unsafe places. As many as slightly over half the youth within the refugee settlements in Uganda have reported at least one or more life time incidence of various forms of violence including physical, emotional and sexual violence [26].

Despite the numerous challenges, support has come in from many places for these CA-HIV. For starters, the HCWs have provided care though this has not necessarily been in line with national guidelines for example when it came to use of mental health screening instruments. Possible reasons for this may include lack of training, unclear procedures and lack of follow up [27]. YAPs (peer workers) were reported to be doing a good job in talking to children and adolescents and following up on them. Indeed, peer health workers have been reported to have a pivotal role in HIV care among young people [28].

The role of the HCWs was tremendously supported by many partners as is usually the case in many refugee settings. Unfortunately, the partners’ services often suffer from a discomforting discontinuity once their projects are terminated. This could be because the partners often focus on getting refugees out of the immediate emergency as opposed to supporting them to achieve self-sustenance [29]. Notably, more sustainable methods have been advocated for by other scholars – particularly embedding partners’ practices into existing public service structures with a program as opposed to a project operations mentality [30]. Besides the specialized personnel is the role of parents and families who are key to HIV care among affected children. These have been known to play a crucial role in HAART adherence, psycho-social support, education and economic security [31]. Finally, self-reliance and resilience in the form of special skills, sports, community groups and peer support was noted and this is a window of opportunity for future MHPSS interventions in this population.

### Limitation

In this study, children with HIV and their caretakers were selected from health center attendance records, meaning that those inconsistently engaged in formal HIV care and their experiences were not included in this study. To mitigate this, we included diverse perspectives from service providers (healthcare workers, teachers, police officers, and refugee welfare council members) who encounter children at various stages of HIV care, including those who are inconsistently engaged. Their insights helped represent the experience of all the CA-HIV to a reasonably acceptable extent.

Pertaining ethical considerations, there is a lot of stigma around mental health issues, therefore, during FGDs, participants may not have been open enough about their experiences with MHPSS. We therefore employed multiple data collection methods (FGDs and IDIs), with IDIs allowing for private, confidential exploration of sensitive issues that participants might have been reluctant to discuss in groups. The principal investigator, being a psychiatrist, was trained in empathetic, non-judgmental interviewing techniques and provided brief psychological first aid when distress emerged, fostering trust and openness. Participants were also reminded that their participation was voluntary and that they could skip any question or withdraw at any time.

Developmental considerations posed inherent challenges. The CA-HIV in our study spanned different cognitive and emotional developmental stages. Younger children, in particular, may have had difficulty articulating abstract concepts such as mental health, stress, or emotional well-being, potentially leading to underreporting or overly simplified responses. To address this, we used age-appropriate, simple language during interviews and FGDs and supplemented CA-HIV data with perspectives from caretakers and service providers who could elaborate on seemingly abstract concepts.

Reliance on caretakers for recruitment and information introduced potential biases. Caretakers served as gatekeepers, and those who consented to participate may have been more engaged or supportive of the children in their care. Conversely, children with unsupportive or overwhelmed caretakers (who may face more severe MHPSS challenges) could have been less likely to participate. Furthermore, some children participated in FGDs in proximity (though out of earshot) to their caretakers, which may have influenced their willingness to disclose sensitive information. To mitigate this, we conducted interviews with children separately from their caretakers, providing a private space for disclosure. We also explicitly assured all participants of confidentiality and emphasized that there were no right or wrong answers. The inclusion of service provider perspectives further helped balance any caretaker-related biases by providing independent observations.

Despite these limitations, our use of multiple data sources (CA-HIV, caretakers, and service providers), method triangulation (FGDs and IDIs), and detailed contextual description support the credibility of our findings.

## Conclusion

MHPSS is a broad concept as elaborated by the varying cadres in our study. Efforts should thus be made to establish a common understanding between the varying parties, to allow the issues to be addressed. All factors notwithstanding, MHPSS challenges are contextual and may require economic and several livelihood considerations, the interventions should reflect this. Also, MHPSS interventions as reported by the beneficiaries and implementers are multifaceted involving specialized, non-specialized personnel and the children themselves. It is thus imperative that interventions are availed at the different levels but with special attention to the refugee CA-HIVs, this subpopulation is often subsumed and lost under larger titles like refugees and children.

### Recommendations

**For Policy:** Developing context-specific MHPSS guidelines for refugee CA-HIV is critical. In as much as Uganda's consolidated HIV guidelines provide for MHPSS, specifying for humanitarian settings will go a long way. This will strengthen the integration of mental health into HIV services. Beyond guidelines, ensuring sustainable funding for food and livelihood support is critical considering the prominence of food insecurity as reported in this study. Where applicable, low cost interventions like community sensitization should be promoted to address challenges like stigma.

**For Practice:** Non-specialized MHPSS service providers (teachers, police officers, Refugee Welfare Council members, and community health workers) play a critical role as frontline responders to CA-HIV. Having them sufficiently trained on pertinent issues of MHPSS will greatly enhance the service providers’ human resource base available to support these children. In line with this, young people and adolescent peer supporters (YAPs) should be trained and engaged more systematically. Families were a critical source of support for CA-HIV, family-centered approaches should thus be emphasized.

**For future research:** This exploratory study identified significant MHPSS challenges, but literature on the prevalence of specific mental disorders (like depression, anxiety, PTSD, suicidality) among refugee CA-HIV remains scanty. Future research should aim to cover this gap. Further still, in as much as we had several partners implementing various interventions, effectiveness of many of these remains a grey zone – and would benefit greatly from further study. Research should include cost-effectiveness analyses to inform resource allocation. Other populations that would greatly benefit from further study are the CA-HIV who are out of care as we did not interact with these.
